# Characterization of the Exometabolome of *Nitrosopumilus maritimus* SCM1 by Liquid Chromatography–Ion Mobility Mass Spectrometry

**DOI:** 10.3389/fmicb.2021.658781

**Published:** 2021-07-01

**Authors:** Kai P. Law, Wei He, Jianchang Tao, Chuanlun Zhang

**Affiliations:** ^1^SUSTech Academy for Advanced Interdisciplinary Studies, Southern University of Science and Technology, Shenzhen, China; ^2^Shenzhen Key Laboratory of Marine Geo-Omics Research, Southern University of Science and Technology, Shenzhen, China; ^3^Department of Ocean Science and Engineering, Southern University of Science and Technology, Shenzhen, China; ^4^Southern Marine Science and Engineering Guangdong Laboratory (Guangzhou), Guangzhou, China; ^5^Shanghai Sheshan National Geophysical Observatory, Shanghai, China

**Keywords:** *Thaumarchaeota*, *Nitrosopumilus maritimus*, exometabolome, dissolved organic matter, ion mobility mass spectrometry

## Abstract

Marine *Thaumarchaeota* (formerly known as the marine group I archaea) have received much research interest in recent years since these chemolithoautotrophic organisms are abundant in the subsurface ocean and oxidize ammonium to nitrite, which makes them a major contributor to the marine carbon and nitrogen cycles. However, few studies have investigated the chemical composition of their exometabolome and their contributions to the pool of dissolved organic matter (DOM) in seawater. This study exploits the recent advances in ion mobility mass spectrometry (IM-MS) and integrates this instrumental capability with bioinformatics to reassess the exometabolome of a model ammonia-oxidizing archaeon, *Nitrosopumilus maritimus* strain SCM1. Our method has several advantages over the conventional approach using an Orbitrap or ion cyclotron resonance mass analyzer and allows assignments or annotations of spectral features to known metabolites confidently and indiscriminately, as well as distinction of biological molecules from background organics. Consistent with the results of a previous report, the SPE-extracted exometabolome of *N. maritimus* is dominated by biologically active nitrogen-containing metabolites, in addition to peptides secreted extracellularly. Cobalamin and associated intermediates, including α-ribazole and α-ribazole 5′-phosphate, are major components of the SPE-extracted exometabolome of *N. maritimus*. This supports the proposition that *Thaumarchaeota* have the capacity of *de novo* biosynthesizing cobalamin. Other biologically significant metabolites, such as agmatidine and medicagenate, predicted by genome screening are also detected, which indicates that *Thaumarchaeota* have remarkable metabolic potentials, underlining their importance in driving elemental cycles critical to biological processes in the ocean.

## Introduction

In the ocean, phytoplankton release a variety of biomolecules, from carbohydrates, lipids, and nucleic acids to peptides and proteins, into their surrounding seawater, and those compounds serve as substrates to vast heterotrophic microbial populations underlying the microbial loop in the ocean (Thornton, 2014). Both passive diffusion and active transportation are involved (see Figure 6 in Thornton, 2014), but an overflow metabolism has also been proposed as the major mechanism to account for the exudation of by-products as well as the metabolic intermediates extracellularly (Paczia et al., 2012). The complete set of these extracellular biomolecules is collectively referred to as the exometabolome. Exometabolites released by marine plankton become an integral part of dissolved organic matter (DOM) in seawater and mediate intra- and interspecies microbial interactions. Study of the extracellular metabolites thus allows a direct characterization of the molecular interactions between microbes and their environment by profiling the types of molecules cellular organisms secrete (Douglas, 2020).

Decades of research by marine organic geochemists have revealed a wealth of information regarding the age and chemical characteristics of marine DOM. Still, the identity and the biological functions of individual DOM constituents remain largely unknown despite improvements in enrichment and analytical technologies (Mopper et al., 2007; Nebbioso and Piccolo, 2013; Stubbins and Dittmar, 2014). A major challenge is the short half-lives (on the order of minutes) and concentrations (in the picomolar range) of metabolically active or “labile” DOM compounds in the seawater (Moran et al., 2016), making it extremely difficult for analyzing these compounds. Furthermore, bulk seawater analyses are not able to link specific components to definite biological sources; thus, the relationship between marine plankton diversity and DOM composition remains to be delineated.

Several recent reviews have highlighted the need to reexamine DOM at the molecular level and link DOM compounds to their biological sources, to identify the roles of different microbial groups in DOM cycling (Dittmar and Paeng, 2009; Hansell et al., 2009; Kujawinski, 2011). With the recent advances in cultivation techniques and metabolomic methodology (Kido Soule et al., 2015; Longnecker and Kujawinski, 2016, Longnecker and Kujawinski, 2017), molecular-level investigations of the exometabolome of marine planktons have been reported, including cyanobacteria (Barofsky et al., 2009; Baran et al., 2011; Fiore et al., 2015), diatoms (Longnecker et al., 2015b), and eukaryotic algae (Alsufyani et al., 2017). The exometabolome of marine archaeoplankton and their contributions to the DOM pool, however, has been understudied.

Archaea, once thought to thrive only in extreme environments, are ubiquitous and abundant in the ocean and play pivotal roles in both the global carbon and nitrogen cycles (Santoro et al., 2019). Planktonic marine archaea are now classified into four major groups, marine group I (MGI) (DeLong, 1992; Fuhrman et al., 1992), MGII (Liu et al., 2017; Xie et al., 2018), MGIII (Fuhrman and Davis, 1997), and MGIV (López-García et al., 2001), based on their 16S rRNA genes. MGII − MGIV are all known as heterotrophs (Zhang et al., 2015; Santoro et al., 2019; Martijn et al., 2020), whereas MGI *Thaumarchaeota* (Brochier-Armanet et al., 2008) has been recognized as chemolithoautotrophs, capable of utilizing ammonia as the energy source (Könneke et al., 2005) and playing an important role in primary production in deep-sea marine ecosystems (Zhong et al., 2020). Although *Thaumarchaeota* constitute 20–40% of the ocean’s prokaryotic plankton (Karner et al., 2001), few studies have investigated the contributions of marine chemolithoautotrophic archaea to marine DOM despite their roles in sunlight-independent marine ecosystems.

There has only been one report that examined the exometabolome of marine *Thaumarchaeota*, using flow-injection analysis with ultrahigh-resolution mass spectrometry (UHRMS) (Bayer et al., 2019), which showed that the exudes of three planktonic thaumarchaeotal strains contain a suite of organic compounds dominated by nitrogen-containing compounds. The ions assigned with formulae were classified chemically by van Krevelen diagrams into seven classes of compounds, such as fatty acids and phenols/polyphenols. Although the van Krevelen diagram is one of the most frequently used graphical representations for complex organic mixtures, the assignments of detected formulae are only approximately categorized into several classes of organic compounds based on their H:C and O:C ratios (Kim et al., 2003). Furthermore, there are large overlaps between compound categories in the van Krevelen diagram, often leading to incorrect or ambiguous compound classification (Rivas-Ubach et al., 2018). Overlaps between compound categories arise from the fact that it is not the molecular formula of a molecule, but its chemical structure, that dictates its chemical classification (Brockman et al., 2018). Also, the O:C and H:C boundaries defining a specific compound category in a van Krevelen diagram substantially differ among published works (D’Andrilli et al., 2015) and have never been accurately defined for robust overall classification of compounds. In contrast, matching the mass spectral characteristics of an unknown molecule with the spectral information in metabolite databases, or further projected with *in silico* fragmentation, has become a widespread approach in untargeted metabolomics (Longnecker et al., 2015a; Barbosa and Roque, 2019; Heal et al., 2019). Although such an approach is not impeccable to errors and restrained by the number and accuracy of metabolites available in the databases and yields only candidate compounds for further verification with authentic standards, it is still likely to give a set of more rectifiable annotations or classifications than by using a van Krevelen diagram. The confidence of spectral annotation can be further enhanced with an inclusion of an evidence-based quantitative score (Schrimpe-Rutledge et al., 2016), via parallel measurements of orthogonal spectral characteristics, such as exact mass, elemental composition, fragmentation pattern, chromatographic retention time, and gas-phase ion mobility.

Ion mobility mass spectrometry (IM-MS) has emerged as one of the most valuable analytical techniques for metabolomics and lipidomics (Kliman et al., 2011; Paglia et al., 2015; Kyle et al., 2016; Paglia and Astarita, 2017; Sinclair et al., 2018). The technique has several advantages over conventional single-dimension MS analyses. Molecular-level structural information in the form of ion-neutral collision cross sections (CCSs) can help characterize the target compound and differentiate conformational isomers. Different chemical classes of compounds can be differentiated via the correlation trend lines. The technique further increases peak capacity and reduces chemical noise, and it is compatible with chromatography techniques. Several groups of researchers have already started exploring the IM-MS technology for DOM analysis, with and without coupling to chromatography (Gaspar et al., 2009; Lu et al., 2018; Tose et al., 2018; Gao et al., 2019; Leyva et al., 2019). While the adoption of IM-MS is still in its early stage, the technology has already shown its promises. Two independent studies specifically aimed to segregate and distinguish isomers in riverine and coastal DOM (Lu et al., 2018) or freshwater wetlands (Leyva et al., 2019) have estimated that the average number of structural and conformational isomers was 6–10 isomers per chemical formula, and this number was substantially smaller than that estimated previously by statistical modeling (Zark et al., 2017).

This research builds upon the findings of Bayer et al. (2019) with an improved analytical protocol for complex biological systems. We aimed to survey the exometabolome of a model chemolithoautotrophic archaeon (*N. maritimus* strain SCM1). Our approach made use of the advanced techniques of IM-MS and corresponding bioinformatics to facilitate the investigation, adhering to the principles and reporting standard of untargeted metabolomics. The results reported herein reveal previously unidentified candidate compounds and provide valuable information for better evaluating the contributions of ammonia-oxidizing archaea (AOA) to oceanic DOM.

## Materials and Methods

### Materials

The following reagents were used for the preparation of synthetic Crenarchaeota medium (SCM). Biotin and pyridoxine dihydrochloride were purchased from Sangon Biotech (Shanghai, China). Calcium chloride dihydrate, HEPES, sodium hydroxide, sodium bicarbonate, potassium phosphate monobasic, FeNaEDTA, nickel(II) chloride hexahydrate, copper(II) chloride dihydrate, 4-aminobenzoic acid, streptomycin sulfate, and ammonium chloride were purchased from Shanghai Macklin Biochemical (Shanghai, China). Sodium chloride, magnesium sulfate heptahydrate, magnesium chloride hexahydrate, potassium bromide, boric acid, manganese(II) chloride tetrahydrate, cobalt(II) chloride hexahydrate, zinc sulfate heptahydrate, nicotinic acid, vitamin B_12_, and vancomycin were purchased from Aladdin Bio-Chem Technology (Shanghai, China). Disodium hydrogen phosphate dodecahydrate was purchased from Sinopharm Chemical Reagent (Beijing, China). Sodium molybdate dihydrate was purchased from Damao Chemical Reagent (Tianjin, China). Calcium pantothenate and thiamine were purchased from Shanghai Jinsui Biochemical (Shanghai, China). Hydrochloric acid was purchased from Dongguan Dongjiang Chemical Reagent (Dongguan, China). Catalase, sodium phosphate monobasic dihydrate, and amphotericin B were purchased from Sigma (Shanghai, China). Solvents and buffers used for sample extraction and mass spectrometry analyses were purchased from Merck (hyper-grade methanol, LC-MS grade formic acid, and LC-grade tert-butyl methyl ether) or Fisher Chemical (optima grade methanol, acetonitrile, and 2-propanol). All reagents and solvents were used without further purification.

### Culture Conditions

**FIGURE 1:**
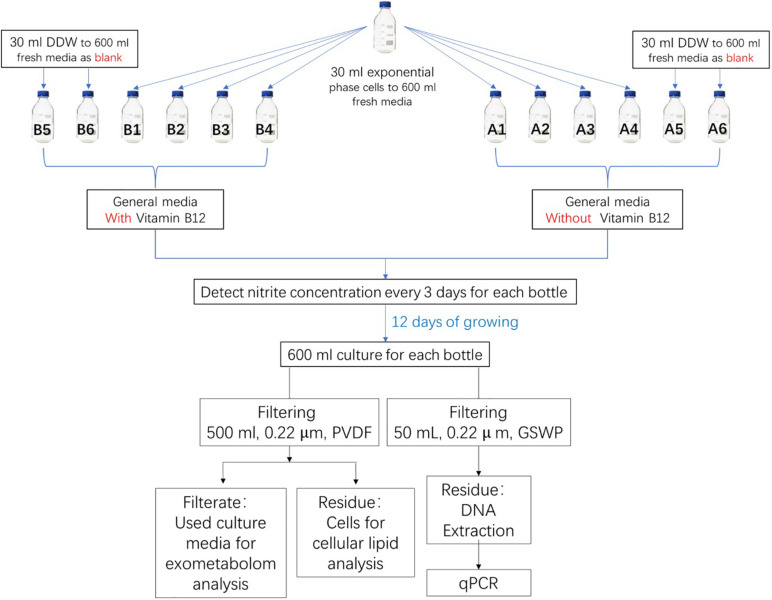
Experimental design for metabolite analysis of *N. maritimus* SCM1. One group of the cultures was prepared with a supplement of vitamin B_12_ and the other group without. Cell growth was monitored by nitrite concentration in the medium. After 15 days of culture, cells were harvested by filtering 500 ml of culture solution. The used and filtered culture medium was also collected, and the organic compounds in the medium were extracted with SPE. The other 50 ml of culture medium was used for qPCR analysis.

*N. maritimus* strain SCM1 was cultivated in HEPES-buffered SCM (pH 7.5, 1 mM NH_4_Cl) as previously described in Könneke et al. (2005) and Martens-Habbena et al. (2009) with an addition of 1 mg/ml catalase solution. To prevent bacterial and organic contaminations, all culture flasks were pretreated with diluted acid and ultrapure water carefully. All cultures were supplemented with 100 μg/ml streptomycin sulfate (Shanghai Macklin Biochemical) to prevent bacterial growth. Cultures were further supplemented with 1 μg/ml vancomycin (Aladdin) and 1 μg/ml Amphotericin B (Sigma) to inhibit the growth of bacteria and fungus. All cultures were supplemented with a mixture of vitamins (4-aminobenzoic acid, thiamine, nicotinic acid, calcium pantothenate, pyridoxine dihydrochloride, and biotin) and non-chelated trace elements (H_3_BO_3_, MnCl_2_, CoCl_2_, NiCl_2_, CuCl_2_, ZnSO_4_, and Na_2_MoO_4_). Two groups of cultures were prepared, but only one group of cultures was prepared with a supplement of 5 μg/ml vitamin B_12_ (Aladdin). Each of these groups was prepared in six 1-l glass bottles, four were biological replica, and two were cell-free controls (Figure 1). Each of the culture bottles was filled with 600 ml of culture medium. Then, 30 ml exponential phase cells was added into four of the bottles, while the other two were added with 30 ml Milli-Q water. All cultures were incubated aerobically at 30°C in a static condition with occasional inspections. The whole cycle of cultivation took approximately 12 days. Cells were harvested by filtering through a 0.22-μm polyvinylidene fluoride (PVDF) filter (Whatman) after reaching the stationary phase. Filters were stored at −80°C until extraction. The liquid medium was collected and was subjected to solid-phase extraction (SPE) on the same day before storage at −80°C.

### Nitrite Detection

Cell growth was monitored by nitrite production. Nitrite concentration was determined using a diazo-colorimetry with photometric detection at 545 nm. In brief, sulfanilamide solution was prepared by mixing *o*-phosphoric acid (6 ml), sulfanilamide (0.4 g), and naphthyl ethylenediamine dihydrochloride (0.02 g) with ultrapure water (40 ml). Eighty microliters of cell culture and 200 μl sulfanilamide solution were mixed with 720 μl ultrapure water. Two hundred microliters of the mixture was then transferred to a well of a 96-well plate. After incubating for at least 5 min, the optical density was determined by the absorbance at 545 nm with a Sunrise Microplate Reader (Tecan, Grödig, Austria). Growth curves were constructed based on triplicate measurements of nitrite. Calibration was performed with known concentrations of sodium nitrite solution (0.1, 0.2, 0.4, 0.6, 0.8, and 1 mM).

### Quantitative PCR

The purity of the cultures was monitored by quantitative real-time PCR (qPCR) of archaeal primer ARC787F (ATTAG ATACC CSBGTAGTCC)–ARC1059R (GCCAT GCACC WCCTC T) and bacterial primer BAC338F(ACTCC TACGG GAGGC AG)–BAC805R (GACTA CCAGG GTATC TAATC C) (Yu et al., 2005). Fifty milliliters of cell culture was filtered by a 0.22-μm membrane (Millipore GSWP4700). Membranes were cut into pieces for DNA extraction. DNA was extracted by Fast DNA Spin Kit for Soil (MP Biomedicals, Solon, United States) following the manufacturer’s protocol. The qPCR analyses were performed on a QuantStudio^TM^ Real-Time PCR Instrument (Thermo Fisher Scientific, Singapore). The reaction volume was 10 μl with 5 μl of TB Green Premix Ex Taq^TM^ II (Takara, Beijing, China), 0.2 μl ROX Reference Dye II (Takara, Beijing, China), 0.4 μM of each primer, 3 μl ultrapure water, and 1 μl of template DNA. Thermal cycling consisted of initial denaturation at 95°C for 30 s followed by 40 cycles of denaturation at 95°C for 5 s, annealing at 60°C for 45 s, and extension at 72°C for 60 s (Chen et al., 2020). The data were analyzed by QuantStudio^TM^ Design & Analysis Software ver. 1.5.1.

### SPE Extraction of Exometabolites

About 500 ml of filtered culture medium was acidified to pH 2 (HCl, analytical grade) and extracted on Agilent Bond Elut PPL sorbent cartridges (200 mg, 3 mL). SPE cartridges were installed on an Agilent Vac Elut SPS 24 manifold and conditioned with 3 ml of methanol and 6 ml of acidified ultrapure water (pH 2). After the extraction, cartridges were further rinsed with 25 ml acidified ultrapure water before storage at −80°C. Before the mass spectrometry analysis, cartridges were freeze-dried overnight and eluted with 1.5 ml of methanol. Positive pressure was applied to cartridges to push the residual liquid through the device. The filtrates were collected and volume reduced to dryness using a centrifugal concentrator (RVC 2-18 CDplus, Martin Christ Gefriertrocknungsanlagen, Osterode am Harz, Germany). The dried residue was reconstituted in 300 μl of 50% methanol. Two hundred fifty microliters was transferred to a sample glass vial, and the remaining solution was pooled to prepare a QC sample.

### IM-MS Analysis

IM-MS analysis was conducted on a Waters Synapt G2-S*i* (Waters, Manchester, United Kingdom) coupled to an ACQUITY UPLC System (Waters, Manchester, United Kingdom) equipped with a Waters CORTECS UPLC T3 column (2.1 × 100 mm, 1.6 μm) and a corresponding guard column. The chromatographic conditions were as follows: solvent A was 0.1% formic acid in water, and solvent B was 0.1% formic acid in acetonitrile. The strong wash solvent was acetonitrile. A linear gradient started at 0% of B; increased to 0.1% at 1 min, 10% at 3 min, 50% at 15 min, 99.9% at 20 min; and maintained at 99.9% for further 3 min, before returning to 0% B at 23.1 min. The flow rate was 0.4 ml/min. The column temperature was maintained at 40°C. Three milliliters of the sample was injected into the system.

The IM-MS system was equipped with an electrospray ionization (ESI) source, operated at HDMS and HDMS^*E*^ modes, and controlled via MassLynx software, version 4.2 SCN 983. Before analysis, the system was mass and drift-time (DT) calibrated with 2 μg/μl sodium iodide solution and Waters Major Mix IMS/Tof Calibration Kit for CCS measurements according to the vendor’s instructions. One nanogram per micoliter of leucine-enkephalin was used as the LockSpray solution. The data were acquired in resolution mode with resolving power ≥30,000 at *m/z* 556. The capillary voltage was 2.8 and 2.2 kV in positive and negative modes, respectively. The sample cone was 40 V, and source temperatures were 120 and 110°C in positive and negative modes, respectively. Cone gas was set to 30 l/h. Desolvatization temperatures were 450 and 500°C in positive and negative modes, respectively. Desolvatization gas was 600 l/h. Nebulizer gas was 6.5 bar. Trap DC bias and exit were set to 40 and 3 V, respectively. IMS and transfer wave velocity were 700 and 181 m/s, respectively. Data were acquired from 50 to 2,000 Da, from 0 to 23.5 min. The transfer collision energy was ramped from 30 to 90 V. Scan time was 0.2 s.

### Data Processing and Analysis

UNIFI, ver. 1.9 SR4 (Waters, Manchester, United Kingdom), was used to visualize and perform pairwise analyses (binary compare) of the raw IM-MS spectra (Rosnack et al., 2016). The raw spectra were further processed by Progenesis QI (Non-linear Dynamics, Newcastle upon Tyne, United Kingdom) ver. 2.4 and underwent automatic deconvolution and alignment. Peak picking limits were set to automatic fewer sensitivity with minimum peak width 1.5 s. Retention time limits were set to ignore ions before 2.6 min. Data were normalized to the default method, normalized to all compounds.

Spectral features were annotated against selected metabolite databases, including BioCyc *N. maritimus* SCM1 library (ver. 24.0), HMDB 4.0 (Wishart et al., 2017), LIPID MAP (release 20200916) (Schmelzer et al., 2007), ChEBI (release 20200901) (Hastings et al., 2016), NPAtlas (release 20191201) (van Santen et al., 2019) databases, and MS-DIAL MS/MS spectral library (ver. 15), with precursor and theoretical fragmentation tolerance with a relative mass error of 5 ppm, using the MetaScope algorithm (ver. 1.0.6901.37313). The search against the LIPID MAP database was performed with an in-house CCS library, and the CCS tolerance was set within 2.5%. Furthermore, the data were searched against the online ChemSpider (ver. 1.0.6905) database, with precursor and fragment tolerance with a relative mass error of 5 ppm, isotope similarity 95%, and elemental composition with H, C, N, O, P, S, Cl, Fe, and Co.

Spectral characteristics, including mass errors, isotope similarities, and similarities between experimental and *in silico* fragmentation spectra, were used for accessing the confidence of the assignments, and a quantitative scoring system was used (Creek et al., 2014). The software calculated the similarity of each spectral characteristic and summed them to an overall confidence score (maximum score 60, or 80 if CCS values were available, Supplementary Tables 1–9). Annotations of the spectral features were tentatively assigned from metabolite candidates with an overall score ≥47. Annotations of the spectral features were therefore level 2b (probable structures, when a unique compound matched to the spectral feature) or level 3 (tentative candidates, with two or more isomeric compounds matched to the spectral feature and were indistinguishable by MS^2^ or IMS), based on the revised reporting standards proposed by Metabolomics Standards Initiative (Schymanski et al., 2014). In the latter case, the candidate metabolite predicted by genome screening (BioCyc *N. maritimus* SCM1 library) was preferred.

The experimentally measured CCS values of the putative assigned metabolite candidates were further searched against the experimental or predicted values of the CCSbase database (Ross et al., 2020) to filter out unreliable assignments. Chemical classification of the metabolite candidates was conducted with the ClassyFire web server (Djoumbou Feunang et al., 2016). Multivariate statistical analyses were performed with SIMCA-P ver. 14.1. Processed data was first exported from Progenesis QI into EZinfo 3.0 before importing to SIMCA-P.

## Results

### Cultivations and Assessment of the Purity of the *N. maritimus* Cultures

A cultivation protocol was adopted from literature in the preparation of the cultures of *N. maritimus.* Cell growth was monitored by nitrite concentration, and the results are shown in Supplementary Figure 1A. The growth of *N. maritimus* supplemented with vitamin B_12_ (group B) was not significantly different from the group without (group A). After 10 days of cultivation, the concentration of nitrite reached a maximum. Harvesting was attained at the 12 days of cultivation. The purity of the cultures was assessed by qPCR, and results are shown in Supplementary Figure 1B. Our cultures were high purity (98–99%), but the existence of bacteria (∼10^5^ copies/ml) could not be excluded despite our antiseptic measures, the effort of purification, and the use of antibiotics.

### Pairwise Analyses of the Chemical Composition Between the Experimental and Control Media

The experimental culture media of *N. maritimus* and the cell-free control media were extracted by SPE, and the filtrates were analyzed by reverse-phase liquid chromatography coupled to an ion mobility mass spectrometry (RPLC-IM-MS) under positive and negative ionization modes. Since the previous work (Bayer et al., 2019) was performed in negative ionization mode, attention is paid to the data acquired under the negative ionization mode in the following sections and compare our results with those previously reported.

**FIGURE 2:**
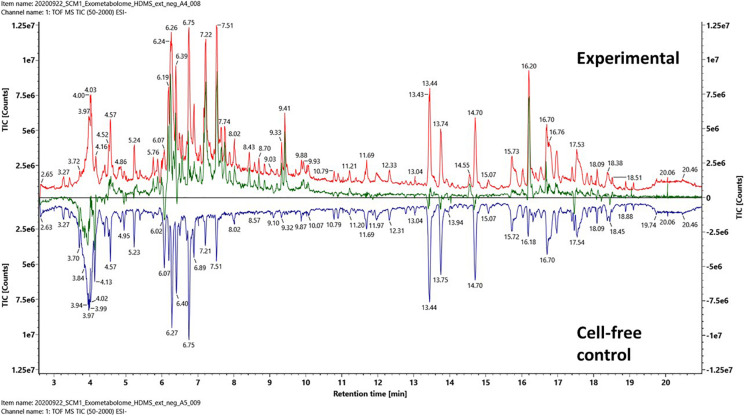
Pairwise comparison analysis of the total ion chromatography (TIC) of an experimental culture medium (red) and a cell-free control culture medium (blue) after PPL-SPE extraction. The net spectrum between the experimental culture medium and cell-free control culture medium is shown as a subtracted chromatography (green) that represents the SPE retained fraction of the exometabolome of *N. maritimus*. Data were acquired in the negative ion mode.

The total ion chromatography (TIC) and IM-MS spectra of the extracted media were manually inspected under UNIFI. TICs of an experimental culture medium and a cell-free control medium are shown in Figure 2. Both chromatographs were complex, which meant not only the experimental medium contained a rich mixture of organics but also the cell-free control medium. The pairwise analysis revealed the differences between the experimental and control media as a subtracted ion chromatography (green). The position of the subtracted chromatography was mostly above the central line (black), indicating that the differences between the experimental and control media were largely due to biomolecules produced by the *N. maritimus* cells and exported extracellularly to the culture medium.

A few regions of the subtracted chromatography were below the central black line, especially at retention time 4 min. Since both the experimental cultures and the control cultures were incubated under identical conditions, any chemical or thermal degradation over the course of incubation would have been canceled out. While we cannot exclude the possibility that some hydrophilic organics were consumed or degraded (by extracellular enzymes) as a result of cultivation, these differences were most likely because the hydrophilic organics were not strongly retained by the modified styrene-divinylbenzene-based PPL SPE sorbent. Hence, the recovery of hydrophilic compounds was reduced as the complexity of the sample increased. Several other regions below the central line appeared to be ion suppression.

**FIGURE 3:**
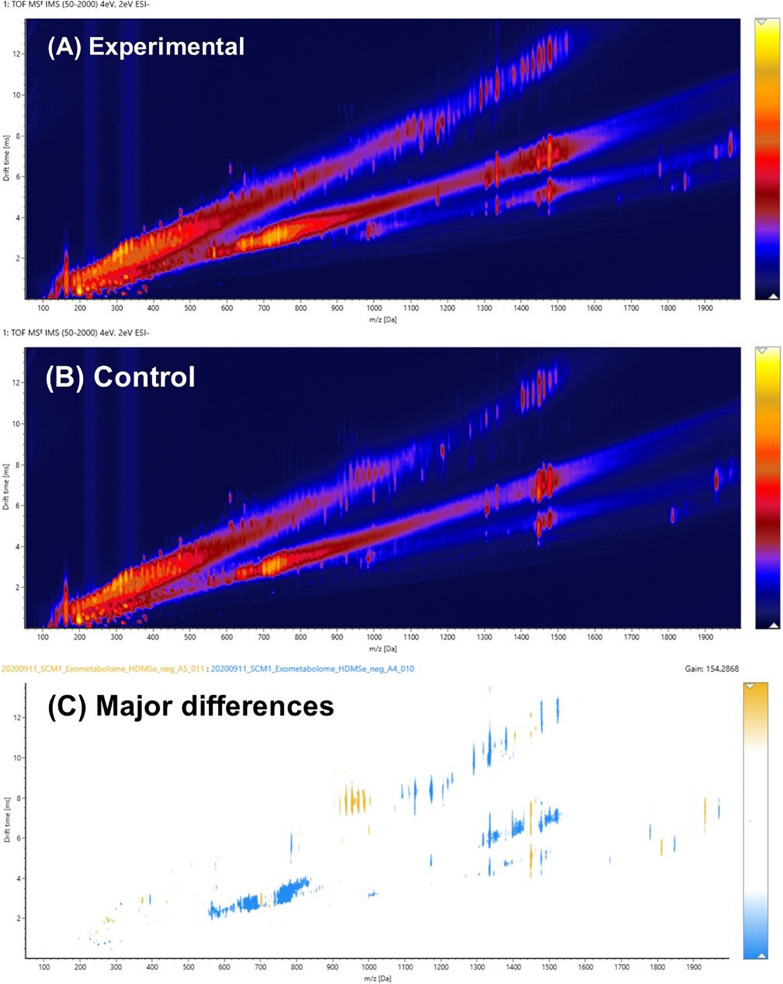
**(A)** Pairwise comparison analysis of the ion mobility conformational spaces of an experimental culture medium and **(B)** a cell-free control medium after PPL-SPE extraction. The major differences between the pair are shown in **(C)**, in which light blue indicates the regions where biomolecules were produced and exported extracellularly to the culture medium, whereas yellow denotes the organics that might have been consumed or degraded over the course of the experiment. Data were acquired in the negative ion mode.

Figures 3A,B were drift time-*m/z* (DT-MZ) plots of an experimental culture medium and a cell-free control medium acquired under negative ion mode. Both plots contain a relatively large number of ion peaks. These ions fell into three major correlation trend lines. Correlation trend lines in ion mobility conformation space typically signify chemical similarity, but their formation can also be because of the charge state of the ions. A pairwise comparison analysis is shown in Figure 3C. The ions with higher intensities in the experimental medium were denoted by blue, whereas the ions with higher intensities in the cell-free control medium were denoted by yellow. The differential ions in Figure 3C equally fell into these three major correlation trend lines.

**FIGURE 4:**
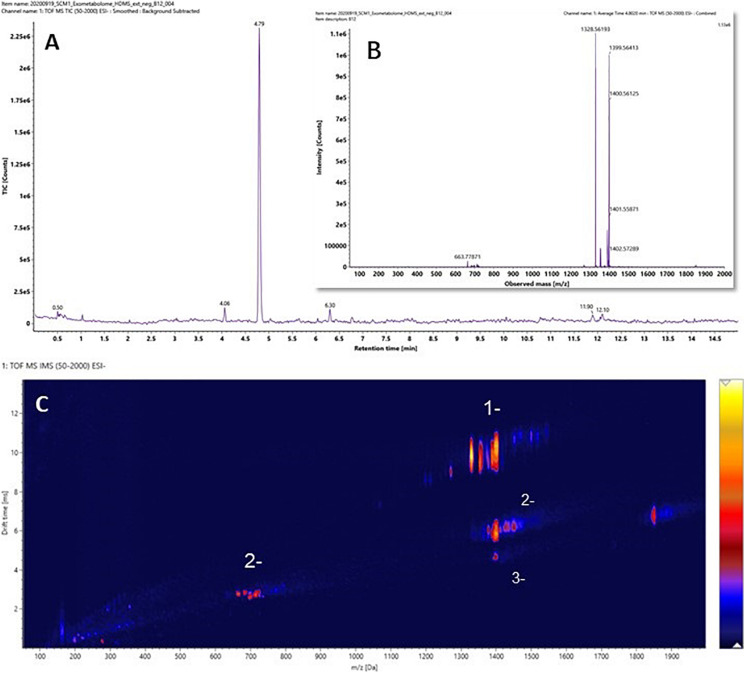
**(A)** The ion chromatography, **(B)** the mass spectrum, and **(C)** the ion mobility characteristics of Vitamin B_12_ (cobalamin). Data were acquired in the negative ion mode.

It was hypothesized that the major differences between the experimental medium and the control medium observed in Figure 3C was mainly due to the biosynthesis and exportation of cobalamins into the culture media. A vitamin B_12_ reference standard was analyzed under the same experimental conditions, and the results are shown in Figure 4. Similar to the unique pattern in Figure 3C, vitamin B_12_ displayed comparable ion mobility characteristics in the conformational space in Figure 4C. These patterns confirmed that one of the major components of the exometabolome of *N. maritimus* was various forms of cobalamin, and perhaps associated intermediates of their biosynthesis.

Although a previous study has identified a complete biosynthetic pathway of cobalamin as a part of porphyrin and chlorophyll metabolism of *N. maritimus* and separated gene clusters encoding distinct stages of cobalamin biosynthesis (Doxey et al., 2015), no experimental result from a cultivation study has been reported. The work previously reported by Bayer et al. (2019) had only scanned a mass window of 92–1,000 Da. As a result, the presence of cobalamin might have been overlooked. Furthermore, their assignments of molecular formula had assumed that ion peaks were singly charged. However, our results have shown that multiply charged ions are also formed in negative ion mode. The observation reported herein was the first experimental evidence of *de novo* biosynthesis of cobalamin in *N. maritimus* and their exportation extracellularly. Furthermore, these observations further demonstrated the potentials of IM-MS in the studies of complex biomolecules produced by marine microorganisms.

### Statistical Analyses of the Experimental and Control Media

The pairwise analyses could only reveal the major differences between a pair of samples. To have a comprehensive view of the exometabolome, both multivariate and univariate statistical methods were employed to differentiate the organic molecules of the exometabolome from the background organics of the control media. A prerequisite of a valid statistical analysis is to have an adequate number of experimental and control replicas. Experiments were therefore carefully designed (Figure 1). Two groups of cultures were prepared: one group of the cultures was supplemented with vitamin B_12_, and the other group without. In each group of the cultures, there were four biological replica and two cell-free controls (a total of 12 samples). Each of these samples was injected three times, along with a QA sample and a pooled QC sample. The whole dataset contained a total of 43 IM-MS runs. The raw spectra were then imported and processed by Progenesis QI and exported to SIMCA-P for further multivariate modeling.

**FIGURE 5:**
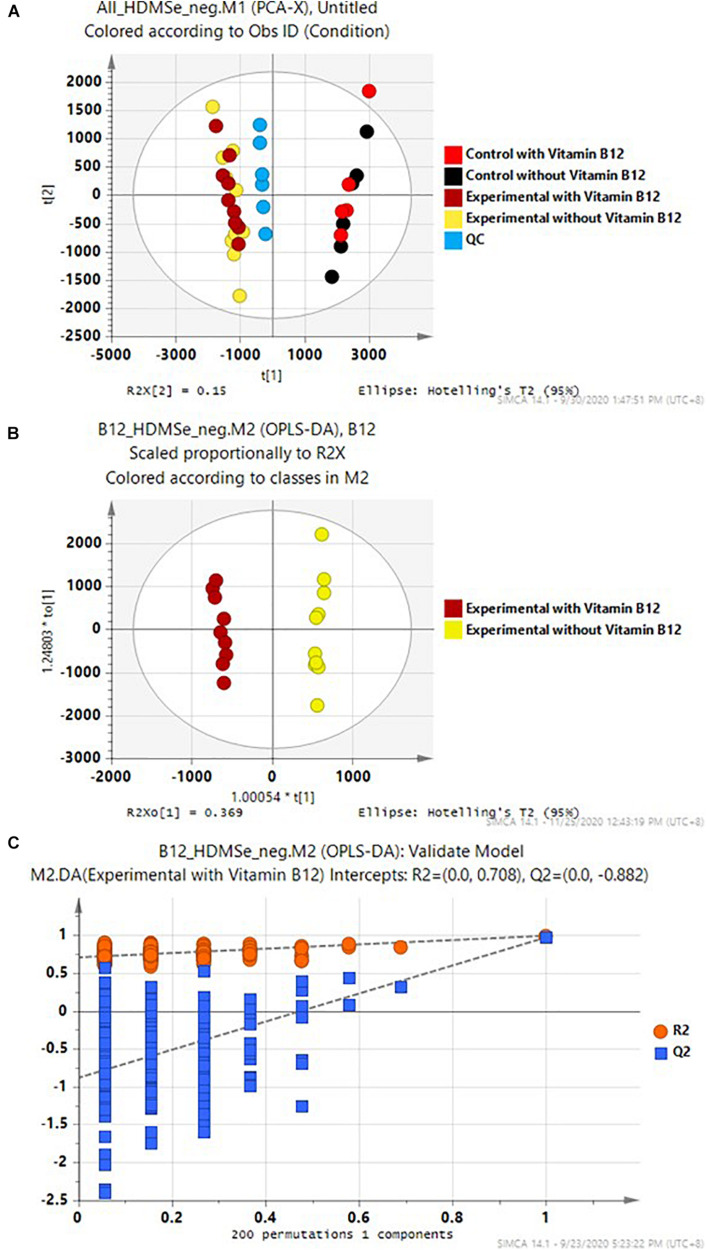
**(A)** PCA of the whole data set, **(B)** OPLS-DA of the experimental media with and without supplement of Vitamin B_12_, and **(C)** 200 permutation tests of the OPLS-DA model in **(B)**.

PCA was employed to provide an overview of the whole dataset. As expected, there were substantial differences between the experimental samples and the cell-free controls. These two classes of samples, regardless of vitamin B_12_ supplement, were well-separated in the PCA score plot (Figure 5A). In contrast, the differences between the cultures with and without vitamin B_12_ supplement were relatively minor, and these two groups did not have aseparation in the PCA score plot. Supervised OPLS-DA was employed to determine the subtle differences between the experimental media with and without supplement of vitamin B_12_ (Figure 5B). The two groups were separated accordingly in the OPLS-DA score plot. This model was further validated with 200 permutation tests (Figure 5C). These results warranted further investigation.

**FIGURE 6:**
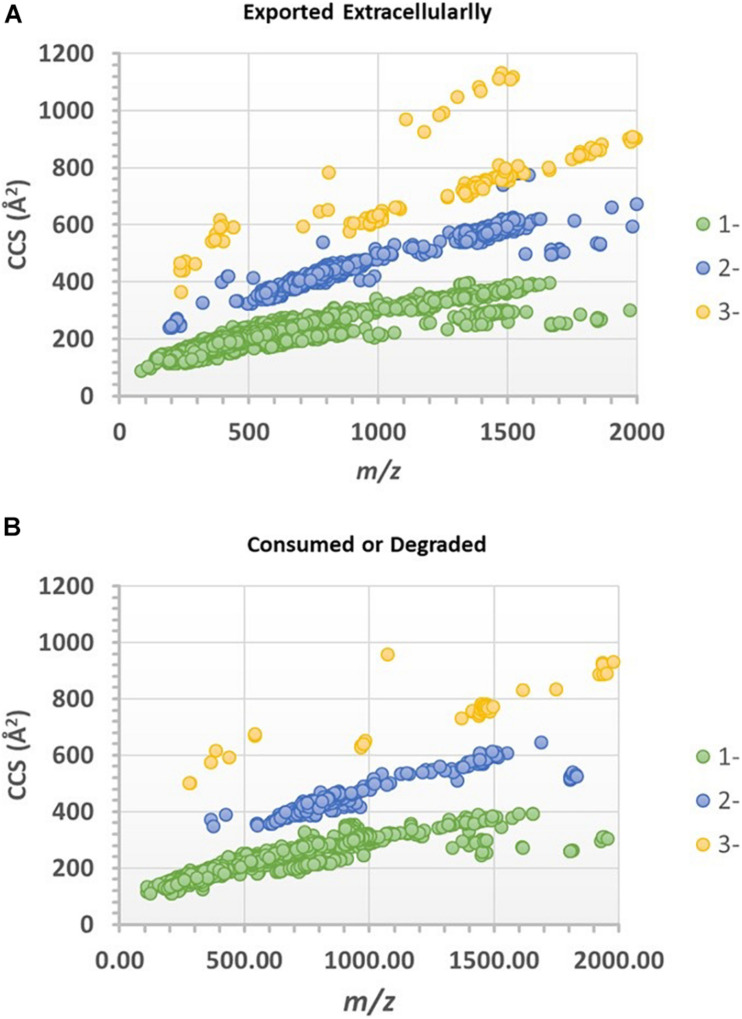
CCS-*mz* plots of the spectral features (ions) that are statistically significantly different between the experimental and control media. **(A)** Features or ions that were found higher in the experimental culture media (assumed exported extracellularly), or **(B)** higher in the cell-free control media (assumed consumed or degraded by extra-cellular enzymes).

Progenesis QI identified a total of 38,643 spectral features after chromatographic alignment and spectral deconvolution. Among these spectral features, 7,227 were found to be statistically significant (ANOVA *p* ≤ 0.01 and had a max fold change ≥ 10) between the experimental and control media (both without vitamin B_12_ supplement). The values of significance level and max fold change were chosen to assure the significant features were not chemical or biological backgrounds, and this was reflected by the *q*-values of false discovery rate analysis (Supplementary Table 1). Of these significant features, 5,569 features were found to be higher in the experimental media relative to the control media, whereas 1,658 features were found to be higher in the control media. The CCS values of these ions were plotted against their *m/z* in Figures 6A,B. Singly, doubly, and triply charged ions were distributed among three major correlation trend lines, similar to that observed in Figure 3, although the order of the correlation trend lines was reversed with the use of CCSs over the drift-time values.

**FIGURE 7:**
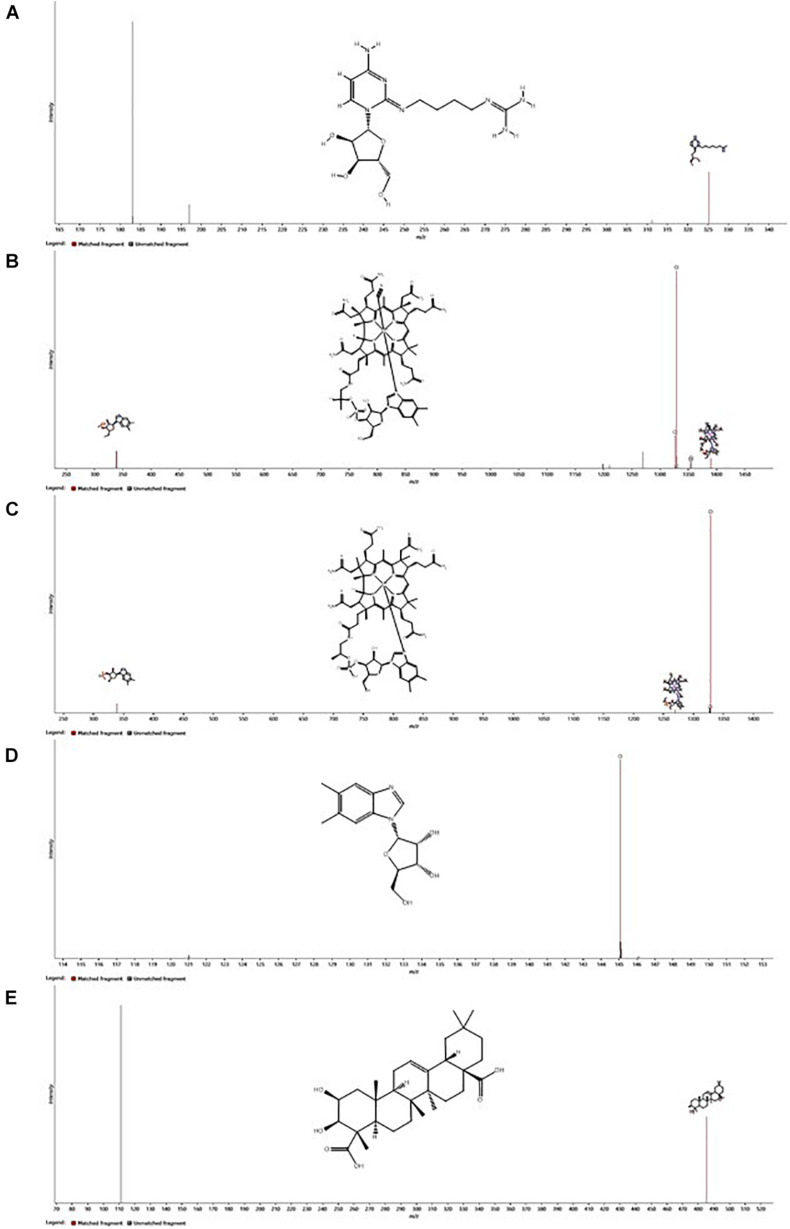
Ion mobility enhanced MS*^*E*^* (HDMS*^*E*^*) fragmentation spectra of the five most important putatively identified metabolites of *N. maritimus*, **(A)** agmatidine, **(B)** cyanocob(III)alamin, **(C)** cob(I)alamin, **(D)** α-ribazole, and **(E)** medicagenic acid, detected from the experimental culture medium in the negative ion mode. Fragment ions consistent with *in silico* fragmentation are highlighted. Inserts show the chemical structures of neutral molecules.

The spectral features that were significantly higher in the experimental media relative to the control media were postulated to be exometabolites produced and exported by the *N. maritimus* cells, and their spectral characteristics were searched against several metabolite databases (see methods), covering from archaeal and bacterial to mammalian metabolites, and were manually assigned tentatively. Seventy features were initially putatively assigned. The CCS measurements of the putative assigned metabolites were further searched against the predicted (or experimental) values modeled by the CCSbase database (Ross et al., 2020). This resulted in filtering out 20 putative assignments. The final set of metabolite candidates comprised of 48 metabolites (Supplementary Table 1) and was chemically classified by ClassyFire (Table 1). The relatively low spectral feature annotation rate was because many of the differential ions were peptides in nature. Among these 48 metabolite candidates, 35 were nitrogen-containing. Five of the identified metabolite candidates have been recognized or predicted to be synthesized by *N. maritimus* or other archaea and have critical biological or ecological roles. These compounds included agmatidine, cyanocob(III)alamin, cob(I)alamin, α-ribazole, and medicagenate. Their HDMS^*E*^ fragmentation spectra are shown in Figure 7.

**TABLE 1:** Chemical classifications and biological roles of the annotated spectral features that are postulated exometabolites of *N. maritimus* SCM1 detected under negative ion mode.

Spectral features	Compound names	Chemical classifications (ClassyFire)	Comments	Archaeal or SCM1 metabolite
2.73_372.0928m/z	DIMBOA glucoside	Carbohydrates and carbohydrate conjugates	A cyclic hydroxamic acid attached to a β-D-glucopyranosyl residue at position 2 via a glycosidic linkage. Plant metabolite involved in benzoxazinoid biosynthesis.	
3.48_555.1140m/z	4,4′-Dihydroxybenzyl sulfone	1-Hydroxy-2-unsubstituted benzenoids	A phenolic plant metabolite found in *Gastrodia elata*.	
3.53_458.1292m/z	1-(2-Hydroxyphenylamino)-1-deoxy-beta-D-gentiobioside 1,2-carbamate	Carbohydrates and carbohydrate conjugates	An aromatic heteropolycyclic plant metabolite.	
3.59_210.0408m/z	Betalamic acid	Amino acids, peptides, and analogs	An alpha-amino acid found in root vegetables.	
3.70_181.0504m/z	Grifolaone A	Furanones	A fungal metabolite from *Grifola frondosa*, a furanone with antimicrobial activity	
3.80_1328.5639m/z	Cob(I)alamin	Corrinoids	A B vitamin produced by archaea. In bacteria, it catalyzes numerous methyl transfer and intramolecular rearrangement reactions.	**✓**
3.88_549.2189m/z	3-Ethyl-2,5-pyrazinedipropanoic acid	Pyrazines	A secondary metabolite produced by a Marine-associated fungus, *Daldinia eschscholzii*	
4.25_362.0991m/z	7-Amino-1-(beta-D-ribofuranosyl)pyrrolo[4,3,2-de]quinolin-8(1H)-one	Pyrroloquinolines		
4.43_277.1181m/z	α-Ribazole	Benzimidazole ribonucleosides and ribonucleotides	An intermediate in riboflavin (vitamin B2) metabolism; involved in porphyrin and chlorophyll metabolism (Vitamin B_12_ biosynthesis).	**✓**
4.59_531.1704n	5′-O-[(Diethoxyphosphoryl)acetyl]-N-isobutyrylguanosine	Purine nucleosides		
4.77_678.3080m/z	Rhizoxin Z2	Terpene lactones	A fungal metabolite from *Rhizopus microsporus*.	
4.78_1354.5671n	Cyanocob(III)alamin	Corrinoids	The most common form of Vitamin B_12_, involved in DNA synthesis and cellular energy production.	**✓**
4.82_531.1705n	5′-O-[(Diethoxyphosphoryl)acetyl]-N-isobutyrylguanosine	Purine nucleosides		
5.08_444.2461m/z	Asn-Leu-Leu-Ser	Amino acids, peptides, and analogs	A tetrapeptide.	
5.14_412.1253m/z	N-[2-(Diethylamino)-2-oxoethyl]-3-(4-methoxyphenyl)-1-pyrrolidinecarboxamide	Amino acids, peptides, and analogs		
5.15_1092.4961m/z	CDP-1,2-diarachidonoyl-sn-glycerol	CDP-glycerols	A mammalian metabolite.	
5.93_633.3250m/z	Withalongolide I	Steroid lactones	A plant metabolite from *Physalis longifolia.*	
7.89_775.4197m/z	(2S,5S,9S,10S,13S,16S,19S)-19,22-Diamino-5-(3-amino-3-oxopropyl)-13-[(2S)-2-butanyl]-9-hydroxy-2-(hydroxymethyl)-10-isobutyl-16-isopropyl-4,7,12,15,18,22-hexaoxo-3,6,11,14,17-pentaazadocosan-1-oic acid	Hybrid peptides	A hybrid peptide that formed as a result of a fusion of amino acid sequences originating from different peptides.	
8.62_358.1865m/z	2-Methoxy-1-(1-methyl-4-{[2-(1-pyrrolidinyl)ethoxy]methyl}-1,4,6,7-tetrahydro-5H-[1,2,3]triazolo[4,5-c]pyridin-5-yl)ethanone	Triazolopyridines		
8.76_274.0380m/z	(2E)-2-[1-(3-Amino-4-nitrophenyl)ethylidene]hydrazinecarbothioamide	Nitrobenzenes		
8.92_460.1394m/z	2-Hydroxy-4-{(1E)-3-[(2S)-2-(hydroxymethyl)-1-pyrrolidinyl]-2-methyl-3-oxo-1-propen-1-yl}phenyl 6-deoxy-alpha-L-galactofuranoside	Carbohydrates and carbohydrate conjugates		
8.94_274.0379m/z	6-Hydroxyphenazine-1-carboxamide	Benzodiazepines	A phenazine metabolites with antimicrobial activities from soil-derived *Streptomyces* species.	
9.04_418.1138m/z	*cis*-Zeatin-O-glucoside	Fatty acyl glycosides	A cytokinin (plant hormone), an intermediate in zeatin biosynthesis.	
9.59_358.1862m/z	2-Methoxy-1-(1-methyl-4-{[2-(1-pyrrolidinyl)ethoxy]methyl}-1,4,6,7-tetrahydro-5H-[1,2,3]triazolo[4,5-c]pyridin-5-yl)ethanone	Triazolopyridines		
10.30_312.0717m/z	Toyocamycin	Pyrrolopyrimidine nucleosides and nucleotides	An *N*-glycosylpyrrolopyrimidine. It is an antimetabolite and induces apoptosis.	
10.82_318.0615m/z	Immucillin G	Pyrrolopyrimidines	A purine nucleoside phosphorylase (PNP) inhibitor.	
11.70_274.0377m/z	(2E)-2-[1-(4-Amino-3-nitrophenyl)ethylidene]hydrazinecarbothioamide	Nitrobenzenes		
12.01_719.3973m/z	Leu-Thr-Gln	Amino acids, peptides, and analogs	A tripeptide.	
12.64_238.0706m/z	(2R,4S)-4-Carboxy-3-(ethoxymethyl)-2-pyrrolidiniumcarboxylate	Amino acids, peptides, and analogs	A D-alpha-amino acid, a proline derivative.	
12.91_252.0867m/z	Deidaclin	Carbohydrates and carbohydrate conjugates	A cyanogenic glycoside.	
12.94_523.3060m/z	Medicagenate	Triterpenoids	A predicted triterpenoid of SCM1.	**✓**
14.57_194.0820m/z	2-(2,6-dihydroxy-3,4-dimethoxycyclohexylidene)acetonitrile	Alcohols and polyols	A predicted bacterial metabolite, produced by the metabolism of 2-(2-hydroxy-3,4-dimethoxy-6-{oxy}cyclohexylidene)acetonitrile.	
14.87_501.2256m/z	3097-C2	1-Hydroxy-2-unsubstituted benzenoids		
15.06_501.2277m/z	2-(2,4-Dihydroxy-5-methoxyphenyl)-3-(3,7-dimethylocta-2,6-dien-1-yl)-5,7-dihydroxy-6-(3-methylbut-2-en-1-yl)-4H-chromen-4-one	Flavones	A predicted flavonoid.	
15.24_264.0865m/z	*N*-Phenylacetylglutamic acid	Amino acids, peptides, and analogs	A glutamic acid derivative.	
15.34_415.1912m/z	Sphingosine 1-phosphate	Phosphosphingolipids	A phospholipid (no ether or ether bond)	
15.54_238.1067m/z	*N*-octanoyl-(2S)-hydroxyglycine	Amino acids, peptides, and analogs	An N-acyl-alpha amino acid.	
16.22_343.2630m/z	5-(2-oxo-Heptadecyl)resorcinol	Benzenediols	A phenolic lipid.	
16.30_238.1438m/z	Petasinine	Pyrrolizidines	A pyrrolizidine from green vegetables.	
17.28_265.0823m/z	1-(2-Deoxy-2-methylene-beta-D-lyxo-hexopyranosyl)-5-methyl-2,4(1H,3H)-pyrimidinedione	Pyrimidines and pyrimidine derivatives		
17.58_521.3107m/z	TG(18:0(11S-acetoxy)/2:0/2:0)	Triacylglycerols	A plant lipid.	
17.81_336.1795m/z	Agmatidine	Pyrimidine nucleosides	A modified cytidine present in the wobble position of the anticodon of several archaeal AUA decoding tRNAs. Agmatidine is essential for correct decoding of the AUA codon in many archaea and is required for aminoacylation of tRNAIle2 with isoleucine.	**✓**
18.14_705.5099m/z	1alpha-hydroxy-23,24,25,26,27-pentanorvitamin D3/1alpha-hydroxy-23,24,25,26,27-pentanorcholecalciferol	Secosteroids	Vitamin D and derivatives.	
18.32_549.3787m/z	Cryptocaryol A	Fatty alcohols	A plant metabolite.	
18.36_517.3526m/z	4-{(1R,3S)-2,2-Dimethyl-3-[(1R,4R,5S)-1-methylbicyclo[2.1.0]pent-5-yl]cyclopropyl}-3-hydroxy-2-butanone	Carbonyl compounds		
18.53_517.3527m/z	Petasitolone	Sesquiterpenoids	A prenol lipid.	
18.58_565.3182m/z	25-Cinnamoyl-vulgaroside	Sesterterpenoids	A cheilanthane sesterterpenoid.	
18.65_441.2639m/z	Ratjadone B	Fatty acids and conjugates	A medium-chain fatty acid.	

*Metabolites associated with archaea or computational predicted to be synthesized by SCM1 (based on BioCyc Nitrosopumilus maritimus, Strain SCM1, ver. 24.0) are marked.*

Agmatidine is a derivative of nucleoside cytidine, in which the 2-keto group on the cytosine ring is substituted by the amino group of agmatine. Although the BioCyc database does not predict the biosynthesis of agmatidine by *N. maritimus*, a group of Japanese scientists has long shown that agmatine is an essential metabolite for the viability of a sulfur-reducing hyperthermophilic archaeon, *Thermococcus kodakarensis* (Fukuda et al., 2008). Subsequent studies have further demonstrated that archaeal cells use agmatine to synthesize 2-agmatinylcytidine of tRNA^*Ile*^, which decodes isoleucine codon AUA specifically and prevents misreading of the methionine codon AUG (Ikeuchi et al., 2010; Mandal et al., 2010).

Cyanocob(III)alamin, cob(I)alamin, and α-ribazole are all participating in cobalamin biosynthesis. The end product is adenosylcob(III)alamin (cobamamide), which is one of the two biologically active forms of vitamin B_12_. α-Ribazole is the lower axial ligand of cobalamin. While being a critical component of cobalamin biosynthesis, no study has ever reported that *N. maritimus* is capable of synthesizing α-ribazole (or its precursor α-ribazole-5-phosphate). The detection of cobalamin and α-ribazole further infers the biogeochemical and ecological roles played by MGI archaea.

Medicagenate (castanogenin) belongs to the class of organic compounds known as oleanane triterpenoids. Oleanane triterpenoids have been isolated from more than 1,600 plant species, including many dietary and medicinal plants (Pollier and Goossens, 2012). Medicagenic acid isolated from the roots of *Herniaria glabra L* exhibits potent fungistatic effects against several plant pathogens and human dermatophytes (Zehavi and Polacheck, 1996; Kozachok et al., 2020). Remarkably, several metabolite candidates, such as DIMBOA-glucoside, 4,4’-dihydroxybenzyl sulfone, 1-(2-hydroxyphenylamino)-1-deoxy-beta-D-gentiobioside 1,2-carbamate, betalamic acid, withalongolide I, *cis*-zeatin-O-glucoside, and cryptocaryol A, have only been isolated from various higher plants.

### Effects of Vitamin B_12_ Supplement

Because of the critical physiological and ecological roles of vitamin B_12_ in *N. maritimus*, we asked a question: what would happen to the composition of the exometabolome if the archaeal cultures were provided with a surplus of vitamin B_12_. The same method was applied to distinguish the metabolites that are statistically significantly different between the culture media supplemented with vitamin B_12_ and those without. The CCS values of these ions were plotted against their *m/z* in Supplementary Figures 2A,B. In total, only 323 spectral features were found to be statistically significant (ANOVA *p* ≤ 0.01 and had a max fold change ≥ 10) between the culture media with and without vitamin B12 supplement. Less than 15 of these spectral features were annotated with metabolite candidates.

In the culture media supplemented with vitamin B_12_, three metabolites were detected with higher intensities along with cyanocob(III)alamin added to the cultures (Table 2 and Supplementary Table 2). They were hydroxocobalamin, cob(I)alamin, and boldine. Hydroxocobalamin, cob(I)alamin, and cyanocob(III)alamin were interchangeable forms of vitamin B_12_ with different upper axial ligands. An increase in their concentration was expected (i.e., not a physiological response because of the stimulus). The detection of boldine was nonetheless an unexpected observation. Boldine is an alkaloid that has only been isolated from higher plants (O’Brien et al., 2006; Han et al., 2008). More importantly, it is an antioxidant that effectively protects against free radical-induced lipid peroxidation or enzyme inactivation. In contrast, the culture media lacking a vitamin B_12_ supplement had higher intensities of oligosaccharides or other carbohydrate conjugates (Table 3 and Supplementary Table 3). The reason for that was unknown.

**TABLE 2:** Chemical classifications of the annotated spectral features that are increased in the culture media with the supplement of Vitamin B_12_.

Spectral features	Compound names	Chemical classifications (ClassyFire)	Comments	Archaeal or SCM1 metabolite
3.80_1390.5645m/z	Hydroxocobalamin	Corrinoids	A member of cobalamins and a precursor of two cofactors or vitamins (Vitamin B_12_ and methylcobalamin).	
4.78_1354.5671n	Cyanocob(III)alamin	Corrinoids	A member of cobalamins and a precursor of cob(I)alamin.	
17.38_362.1157m/z	Boldine	Aporphine	An alkaloid of the aporphine class that can be found in the boldo tree and *Lindera aggregata*. Boldine has antioxidant activity that effectively protects against free radical-induced lipid peroxidation or enzyme inactivation.	

**TABLE 3:** Chemical classifications of the annotated spectral features that are reduced in the culture media with the supplement of Vitamin B_12_.

Spectral features	Compound names	Chemical classifications (ClassyFire)	Comments
2.64_987.3047m/z	β-D-Araf-(1→2)-α-D-Araf-(1→3)-[β-D-Araf-(1→2)-α-D-Araf-(1→5)]-α-D-Araf-(1→5)-α-D-Araf-(1→5)-D-Araf	Oligosaccharides	A branched heptasaccharide comprising seven D-arabinofuranose units
2.77_645.1887m/z	1-[(2-Hydroxy-1-naphthyl)(2-thienyl)methyl]-2-pyrrolidinone	Naphthols and derivatives	
2.78_777.2303m/z	L-α-D-Hepp-(1→2)-L-α-D-Hepp-(1→3)-[β-Glcp-(1→4)]-L-α-D-Hepp	Oligosaccharides	A branched tetrasaccharide consisting of three L-glycero-α-D-manno-heptosyl residues (one at the reducing end) and a single β-D-glucosyl residue.
2.86_909.2725m/z	4-Methoxybenzyl 3-[(4-acetyl-1-piperazinyl)sulfonyl]benzoate	Benzoic acids and derivatives	
2.87_851.2640m/z	α-D-Manp-(1→2)-α-D-Manp-(1→5)-β-D-Araf-(1→2)-α-D-Araf-(1→5)-α-D-Araf-(1→5)-D-Araf	Oligosaccharides	A hexasaccharide composed of two mannopyranose and four arabinofuranose residues in an α(1→2), α(1→5), β(1→2), α(1→5), and α(1→5) linear sequence.
2.91_1041.3150m/z	(5E)-5-(4-Methoxybenzylidene)-3-(4-oxo-2-phenyl-4H-chromen-6-yl)-2-phenyl-3,5-dihydro-4H-imidazol-4-one	Flavones	
2.96_909.2716m/z	5-Methyl-4-[2-(3-methyl-1,2,4-oxadiazol-5-yl)-1H-indol-5-yl]-N-[1-(2-thienyl)ethyl]-1H-imidazole-2-carboxamide	Indoles	
3.01_777.2299m/z	3,4,5-Trihydroxy-6-{[(16S)-5,6,11-trihydroxy-8,8,10,12,16-pentamethyl-3-[1-(2-methyl-1,3-thiazol-4-yl)prop-1-en-2-yl]-9-oxo-17-oxa-4-azabicyclo[14.1.0]heptadec-4-en-7-yl]oxy}oxane-2-carboxylic acid	O-Glucuronides (carbohydrate conjugates)	Belongs to the class of organic compounds known as o-glucuronides. These are glucuronides in which the aglycone is linked to the carbohydrate unit through an O-glycosidic bond.
5.93_633.3250m/z	Withalongolide I	Steroid lactones	A plant metabolite from *Physalis longifolia.*
14.00_397.1103m/z	Propyl 4-[(E)-(2,4-dinitrophenoxy)-NNO-azoxy]-1-piperazinecarboxylate	Piperazine carboxylic acids and derivatives	

### Analyzing the Culture Media in Positive Ion Mode

Consistent with the results of the previous study, the exometabolome of *N. maritimus* was dominated by nitrogen-containing compounds that potentially reflect the fundamental roles of nitrogen metabolism in AOA. Nitrogen-containing compounds tend to form positively charged ions. Henceforth, attention is paid to data acquired in positive ion mode.

The positive TICs of an experimental medium and a cell-free control equally revealed that both media contained a rich mixture of organic compounds (Supplementary Figure 3). The subtracted ion chromatograph was well above the central line. Most compounds were eluted from 3 to 10 min, but a few intense peaks eluded at 16 to 18 min. Similarly, the ions of an experimental culture medium and a cell-free control medium acquired under positive ion mode were distributed into three major correlation trend lines in the DT-MZ plots (Supplementary Figures 4A,B). A pairwise comparison analysis is shown in Supplementary Figure 4C. A comparison with the ion mobility characteristics of vitamin B_12_ (Supplementary Figure 5C) suggested that the major differences between the two media were more than cobalamin alone. These ions or compounds were primarily located at the mass region below *m/z* 800 and might be the most important contributors to marine DOM and hence the oceanic carbon and nitrogen cycles. The previous study had only performed negative ion mode analyses, and these potentially crucial components were unnoticed.

The statistical approach was further exploited to discriminate the differences between the experimental media and the cell-free control media. Over 36,000 spectral features were identified by Progenesis QI. This number of features was larger than expected owning to an extensive in-source fragmentation under positive mode. As shown by the mass spectrum of the vitamin B_12_ reference standard, the positive ion spectrum contained not only ion peaks of various adducts but also a range of in-source fragment ions (Supplementary Figure 5B). It was inevitable that some in-source fragment ions were indistinguishable from molecular ions. Among these spectral features, 8,627 were found to be statistically significant (ANOVA *p* ≤ 0.01 and had a max fold change ≥ 10) between the experimental and control media (both without vitamin B_12_ supplement). Of these significant features, 5,496 features were found to be higher in the experimental media relative to the control media, whereas 3,131 features were found to be higher in the control media. The CCS values of these ions were plotted against their *m/z* in Supplementary Figures 6A,B. Similar to the negative ion data, the differential ions equally fell into three major charge states.

Of the 5,496 features that were found to be higher in the experimental media relative to the control media, 169 features were putatively assigned with metabolite candidates initially. After considering the experimental CCS values with the predicted one, 99 metabolite candidates remained. These compounds were postulated to be exometabolites (Supplementary Table 4) and were chemically classified (Supplementary Table 5). These metabolite candidates consisted of a wide range of organic compounds with varying physicochemical properties. Many of these candidates fell into the category of amino acids, peptides (including depsipeptides), carbohydrates, and carbohydrate conjugates. Several compounds could be identified as lipids. However, few were saturated fatty acids, phenols, or polyphenols. A striking result was the detection of α-ribazole 5′-phosphate, which is one of the predicted metabolites of *N. maritimus* and a product of α-ribazole (detected in negative ion mode). Both are involved in cobalamin biosynthesis. On the other hand, 953 spectral features were found to have significantly altered with or without the supplement of vitamin B_12_ (583 features increased and 370 features decreased). Still, only 23 of these features were annotated with metabolite candidates (Supplementary Tables 6, 8) and chemically classified (Supplementary Tables 7, 9). No specific trend was observed.

## Discussion

This work represents significant progress toward the comprehensive characterization of the exometabolome of marine archaea and their potential interactions with other marine microbes via the DOM pool. Identifying these molecular currencies exchanged within the microbial community may provide key information on microbiome function and its vulnerability to environmental change (Moran, 2015). Our approach explores the potentials of IM-MS with a data-independent acquisition approach (HDMS*^*E*^*) for marine metabolomics. Metabolite identification remains a bottleneck in MS-based metabolomics. We have chosen a method to annotate the spectral features with candidate compounds from selected metabolite databases and assigning candidate metabolites with an evidence-based scoring system. The experimentally measured CCS values were further matched against the theoretical CCS values to verify the metabolites putatively identified. Moreover, a meticulous statistical approach was taken to differentiate the biomolecules released by the cultured cells from the background organics in the culture media.

A noteworthy observation in this study was the identification of cobalamin (vitamin B_12_) and associated metabolites in the exometabolome of *N. maritimus*. The ionic products of these compounds showed a unique pattern in the ion mobility conformational space that could not have been revealed by other mass spectrometry approaches. Cobalamin is a prominent molecular currency and is thought to be synthesized by a subset of bacteria and archaea despite being essential to all forms of life. Producers of cobalamin, therefore, exert a great influence on primary productivity in marine ecosystems. Cobalamin and pseudocobalamin have been identified in seawater in a low pM range (Heal et al., 2014, 2017). Targeted analysis of the cultures of isolated representatives suggested that cobalamin could be mainly produced by certain lineages of heterotrophic alphaproteobacteria and chemoautotrophic *Thaumarchaeota* in the ocean, whereas pseudocobalamin was exclusively produced by marine cyanobacteria, *Prochlorococcus*, and *Synechococcus*.

Cobalamin is biosynthesized via the porphyrin and chlorophyll metabolism (Supplementary Figure 7). These pathways are also involved in biosynthesizing several other biologically important tetrapyrroles, including heme, siroheme, bacteriochlorophyll, and coenzyme F_430_ (Raux et al., 2000). *De novo* biosynthesis of cobalaminoccurs through two alternative pathways: the aerobic or anaerobic routes. A previous study has identified that *N. maritimus* and other *Thaumarchaeota* do not have genes encoding enzymes of the aerobic pathway but genes encoding enzymes specific to the anaerobic pathway (Doxey et al., 2015). Some strains of bacteria and archaea can also synthesize cobalamin by salvaging exogenous corrinoids, although different salvage pathways are used by bacteria and archaea (Escalante-Semerena, 2007). Cobalamin biosynthesis and transportation are regulated by a cobalamin riboswitch in the 5’ untranslated regions (UTRs) of the corresponding genes in bacteria (Fang et al., 2017). In contrast, archaeal genomes have only a sporadic distribution of putative riboswitches like the TPP, FMN, guanidine, lysine, and c-di-AMP riboswitches that are known to occur in bacteria (Angela and Swati, 2019). This may explain that the addition of vitamin B_12_ to the cultures had little or no apparent effect in our experiments.

The structure of cobalamin consists of a cobalt-containing corrin ring, an upper axial ligand, and a lower axial ligand that is tethered to the corrin macrocycle via the nucleotide loop (Supplementary Figure 8). Cobalamin is present across natural systems in several chemical forms that differ in their upper ligand, including the enzymatically active forms of adenosylcobalamin, methylcobalamin, hydroxocobalamin, and the inactivated form cyanocobalamin (Supplementary Figure 9). Different forms of cobalamin are interchangeable and have different upper axial ligands and cobalt oxidation stages. However, much of the cobalamin observed in our study was in the forms of cyanocob(III)alamin and cob(I)alamin due to the mobile phase solvents (water and acetonitrile) and the ionization method used in the experiment.

Cobalamin is a member of a larger family of cobamides that have structural variability in the lower ligand and the nucleotide loop (Sokolovskaya et al., 2020). The lower ligands of cobamides identified thus far belong to three chemical classes: benzimidazoles, purines, and phenolics (Supplementary Figure 10). Unlike the upper ligands, the lower ligands are not exchangeable. CobT enzyme is required to catalyze the activation of the lower ligand base to form an α-ribosylated product. It has shown that the majority of CobT enzymes preferentially attach 5,6-dimethylbenzimidazole (DMB), the lower ligand of cobalamin (Crofts et al., 2013; Hazra et al., 2013).

It has long been established that phytoplankton and bacteria produce a great variety of extracellular substances of varied chemical structures. These substances often play an important physiological role in phytoplankton as well as in ecosystem dynamics (Sañudo-Wilhelmy et al., 2014). Many microorganisms use cobalamin and related cobamides for a variety of metabolic functions, including amino acid metabolism, DNA and RNA synthesis, and carbon and nitrogen metabolism (Sokolovskaya et al., 2020). However, the biosynthesis of cobalamin requires more than 30 enzymatic steps and represents a high genomic and metabolic burden for microbial producers. Most organisms that require cobalamin coenzymes rely on other species to acquire these cofactors. This results in microbial interactions based on cobamide sharing (Seth and Taga, 2014; Sokolovskaya et al., 2020). Since cobalamin and related cobamides play a significant ecological role and are produced exclusively by a subset of bacteria and archaea, it may provide us a means to develop strategies to manipulate microbial communities for human health, agricultural, and environmental applications.

Our results further demonstrated that *N. maritimus* has remarkable metabolic potential and releases a suite of biomolecules extracellularly with various biological and ecological functions, many of which are nitrogen-containing metabolites as well as extracellular peptides/proteins. However, the chemical classification of the set of metabolites detected in this study was in strike contrast to that previously reported (summarized in Table 4). Sugars, amino acid derivatives, fatty acid, phenol, or polyphenol were not detected in negative mode. Fatty acid, phenol, and polyphenol were not detected in positive mode. Although the annotated spectral features represented a fraction of all molecules in the exometabolome, most of these compounds do not fall into the categories or classification conventionally defined by van Krevelen diagram that are not strictly representative of all similar molecules but merely approximate criteria for identifying similarly composed compounds.

**TABLE 4:** Comparing with the observations reported by Bayer et al. (2019) and this study.

Bayer et al. (2019)	This study
Dissolved organic carbon concentrations of solid-phase extracted culture media harvested during late exponential growth was on average two to three times higher than DOC concentrations of medium blanks. This indicated that marine ammonia-oxidizing archaea are not releasing as much (fixed) carbon as compared to heterotrophic bacteria.	The TIC of the solid-phase extracted culture media was not significantly higher than that of the cell-free controls despite 12 days of culturing (early stationary phase). This was likely because archaea are not as metabolic active as bacteria and have a lower metabolic yield.
The exudes of the three planktonic thaumarchaeotal strains contain a suite of organic compounds dominated by nitrogen-containing compounds.	The exometabolome is rich in nitrogen-containing biomolecules, including peptides or proteins, metabolites of a wide range of chemical classes as well as different forms of vitamin B_12_.
The exometabolomes of planktonic thaumarchaea are relatively rich in low-molecular-weight compounds and carboxyl-rich alicyclic molecules (CRAM).	Few or no CRAM was detected.
The exometabolomes of *Nitrosopumilus* contained a high proportion of compounds with molecular formulae characteristic for phenols, polyphenols, highly unsaturated compounds, unsaturated aliphatics, peptides, and saturated fatty acids.	Detected metabolites fell into a wide range of classes varying from carbohydrate conjugates, nucleosides to triterpenoids. Few of the detected metabolites fell into the chemical classification conventionally used by the van Krevelen diagram.
A targeted analysis identified a few ecologically relevant metabolites released by the *Nitrosopumilus* strains, including pantothenic acid (vitamin B_5_) and riboflavin (vitamin B_2_) at lower nM to higher pM concentrations.	Thymidine, pantothenic acid, and riboflavin were not detected.

A limitation of this work was the challenges associated with the extraction of highly hydrophilic metabolites from large volumes of salt-containing aqueous media. While polar functionalized styrene-divinylbenzene polymer-based PPL sorbent is superior SPE resin for extracting dissolved organics in seawater compared to C18 and C8 silica-based sorbents (Dittmar et al., 2008), retention is effective only for non-polar or weakly polar analytes (Johnson et al., 2017). Highly hydrophilic compounds, such as sugars, sugar alcohols, amino acids, and organic acids are not retained by hydrophobic SPE substrate (Sogin et al., 2019) and consequently were not extracted and enriched. Highly water-soluble metabolites, such as osmolytes, ectoine, and hydroxyectoine (Widderich et al., 2016), and β-glutamic acid (Heal et al., 2020), despite being detected previouslyin the intracellular metabolome of *N. maritimus*, were not identified in our experiment. A possible direction is a concurrent use of polymeric PPL and ion-exchange-based SPE substrates for metabolite extraction (Wang et al., 2019), as well as the use of both reversed-phase and hydrophilic interaction liquid chromatography for analysis.

## Data Availability Statement

The original contributions presented in the study are included in the article/ Supplementary Material, further inquiries can be directed to the corresponding author/s.

## Author Contributions

KL designed the study, performed the sample preparation, mass spectrometry, and data analyses, and wrote the manuscript. JT and WH prepared the cell cultures, performed the sample preparation, and conducted the biochemical assays. CZ oversaw the study and edited and approved the final manuscript. All authors contributed to the article and approved the submitted version.

## Conflict of Interest

The authors declare that the research was conducted in the absence of any commercial or financial relationships that could be construed as a potential conflict of interest.
