# Supplementary material for: Characterization of the Exometabolome of Nitrosopumilus maritimus SCM1 by Liquid Chromatography–Ion Mobility Mass Spectrometry
Source: Front Microbiol. 2021 Jul 1;12:658781. doi: 10.3389/fmicb.2021.658781 (PMC8281238; doi:10.3389/fmicb.2021.658781)
Supplement: Supplementary file 1 [file Table_1.DOCX]

Characterization of the Exometabolome of Nitrosopumilus maritimus SCM1 by Liquid Chromatography-Ion Mobility Mass Spectrometry

Supplementary Material

## Index of Supplementary Tables

**Supplementary Table 1:** An export of Progenesis QI that show the annotations of spectral features to metabolites (exometabolome, negative ion mode data). Metabolite assignments are based on overall scores calculated by mass error, fragmentation, and isotope similarity against the metabolites in the databases, and are further checked against the predicted CCS values and verified manually. Anova *p-* and *q-*values are used to determine the significance.

**Supplementary Table 2:** An export of Progenesis QI that show the annotations of spectral features to metabolites (supplement of vitamin B_12_, negative ion mode data). The table is organized in the same way as the Supplementary Table 1.

**Supplementary Table 3:** An export of Progenesis QI that show the annotations of spectral features to metabolites (absence of additional vitamin B_12_, negative ion mode data). The table is organized in the same way as the Supplementary Table 1:

**Supplementary Table 4:** An export of Progenesis QI that show the annotations of spectral features to metabolites (exometabolome, positive ion mode data). The table is organized in the same way as the Supplementary Table 1:

**Supplementary Table 5:** Chemical classifications of the annotated spectral features that are postulated exometabolites of *N. maritimus* SCM1 detected under positive mode. The table is organized in the same way as the Table 1.

**Supplementary Table 6:** An export of Progenesis QI that show the annotations of spectral features to metabolites (supplement of vitamin B_12_, positive ion mode data). The table is organized in the same way as the Supplementary Table 1.

**Supplementary Table 7:** Chemical classifications of the annotated spectral features in the Supplementary Table 6

**Supplementary Table 8:** An export of Progenesis QI that show the annotations of spectral features to metabolites (absence of additional vitamin B_12_, positive ion mode data). The table is organized in the same way as the Supplementary Table 1.

**Supplementary Table 9:** Chemical classifications of the annotated spectral features in the Supplementary Table 8.
