# Supplementary material for: Characterization of the Exometabolome of Nitrosopumilus maritimus SCM1 by Liquid Chromatography–Ion Mobility Mass Spectrometry
Source: Front Microbiol. 2021 Jul 1;12:658781. doi: 10.3389/fmicb.2021.658781 (PMC8281238; doi:10.3389/fmicb.2021.658781)
Supplement: Supplementary file 2 [file Table_2.DOCX]

**A**

**Supplementary Figure 1.** (A) Growth curve of strain SCM1 of *N. maritimus* as measured by nitrite production. Group A was supplemented with Vitamin B12, and group B without. Error bars were the standard deviation of the mean. (B) qPCR assay of the cultures. Purity was defined by the number of archaeal gene copies relative to the total archaeal and bacterial gene copies detected and ranged from 97.17 to 99.27%.

**B**

**A**

**B**

**Supplementary Figure 2.** CCS-*mz* plots of the spectral features (ions) that are statistically significantly different between the mediums of the cultures supplemented with Vitamin B_12_ and those without. (A) Features or ions that were found higher in the mediums of the cultures supplemented with Vitamin B_12_, or (B) higher in the mediums of the normal cultures.


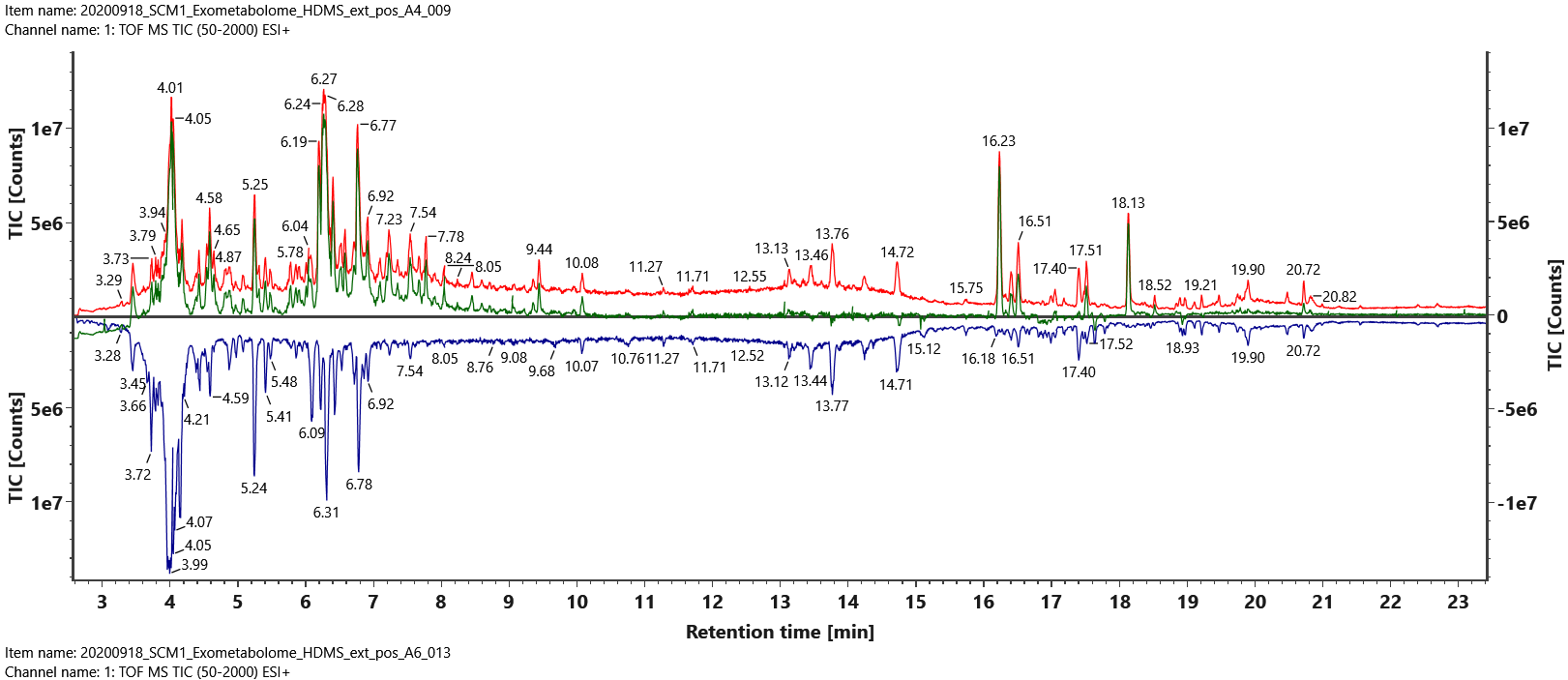


**Cell-free control**

**Experimental**

**Supplementary Figure 3.** Pairwise comparison analysis of the total ion chromatography of an experimental culture medium (red) and a cell-free control culture medium (blue) after PPL-SPE extraction. The differences between the pair are shown as a subtracted chromatography (green) that represents the SPE retained fraction of the exometabolome of *N. maritimus* strain SCM1. Data were acquired in the positive ion mode.


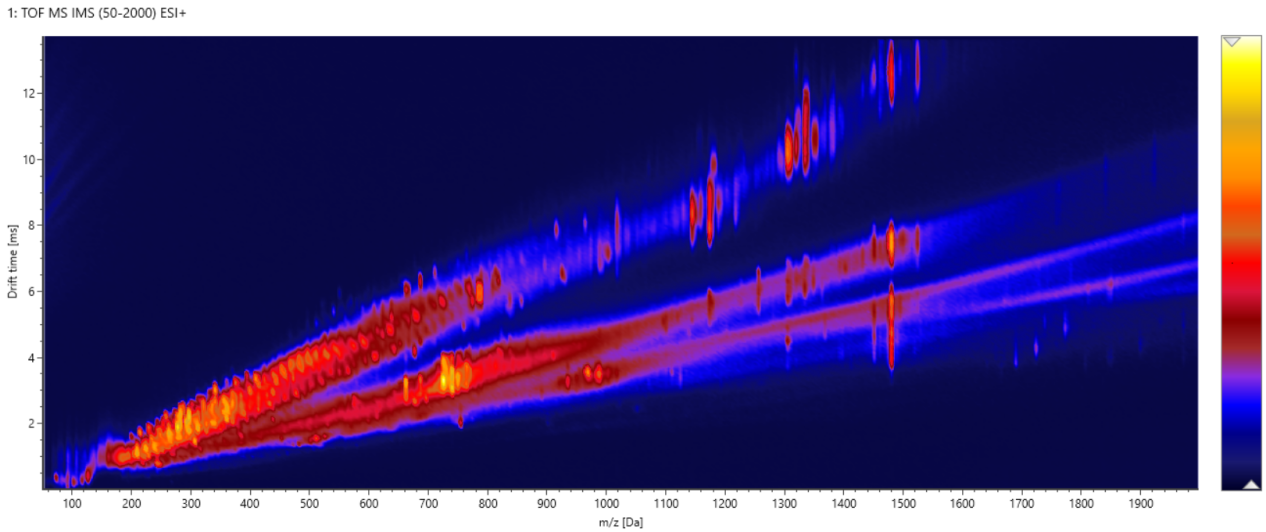

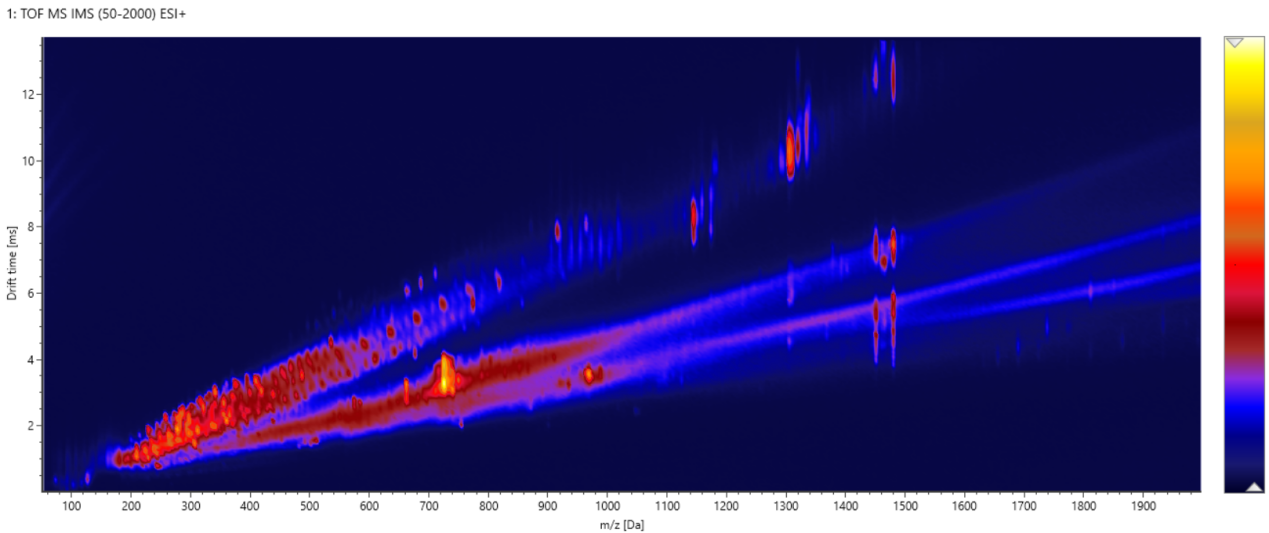


**1+**

**2+**

**3+**

**2+**

**3+**

**1+**

1. **Control**
2. **Experimental**


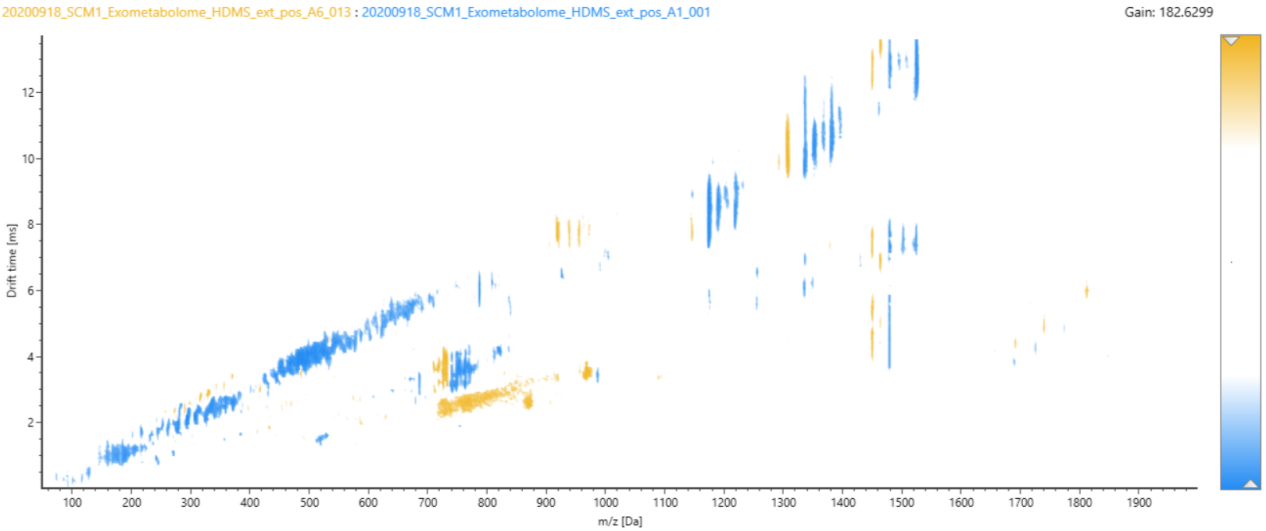


3+

2+

1+

**(C) Major differences**

**Supplementary Figure 4.** (A) Pairwise comparison analysis of the ion mobility conformational spaces of an experimental culture medium, and (B) a cell-free control medium after PPL-SPE extraction. The major differences between the pair are shown in (C), in which light-blue indicates the regions, where bio-molecules were produced and exported extra-cellularly to the culture medium, whereas the yellow denotes the organics that might have been consumed or degraded over the course of the experiment. Data were acquired in the positive ion mode.


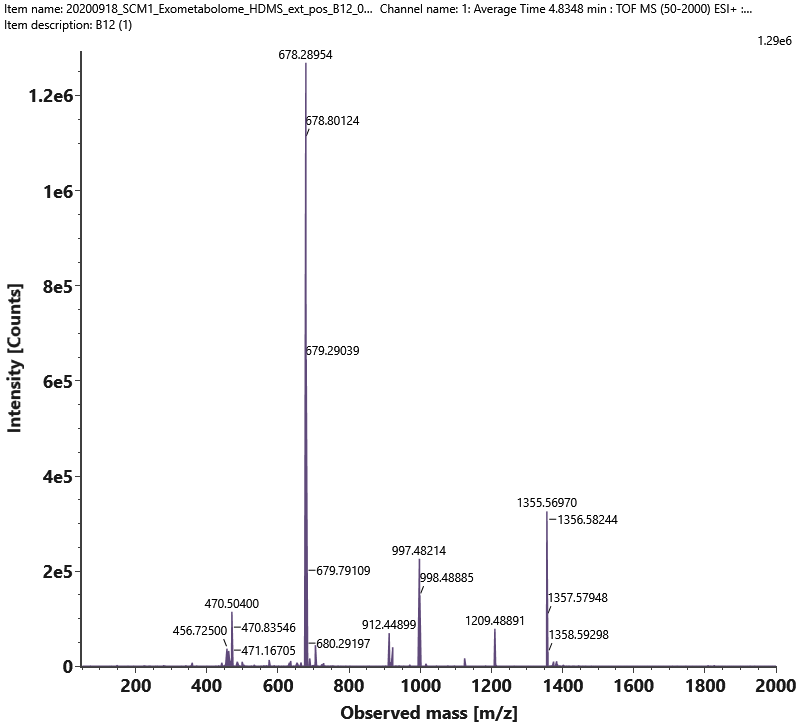

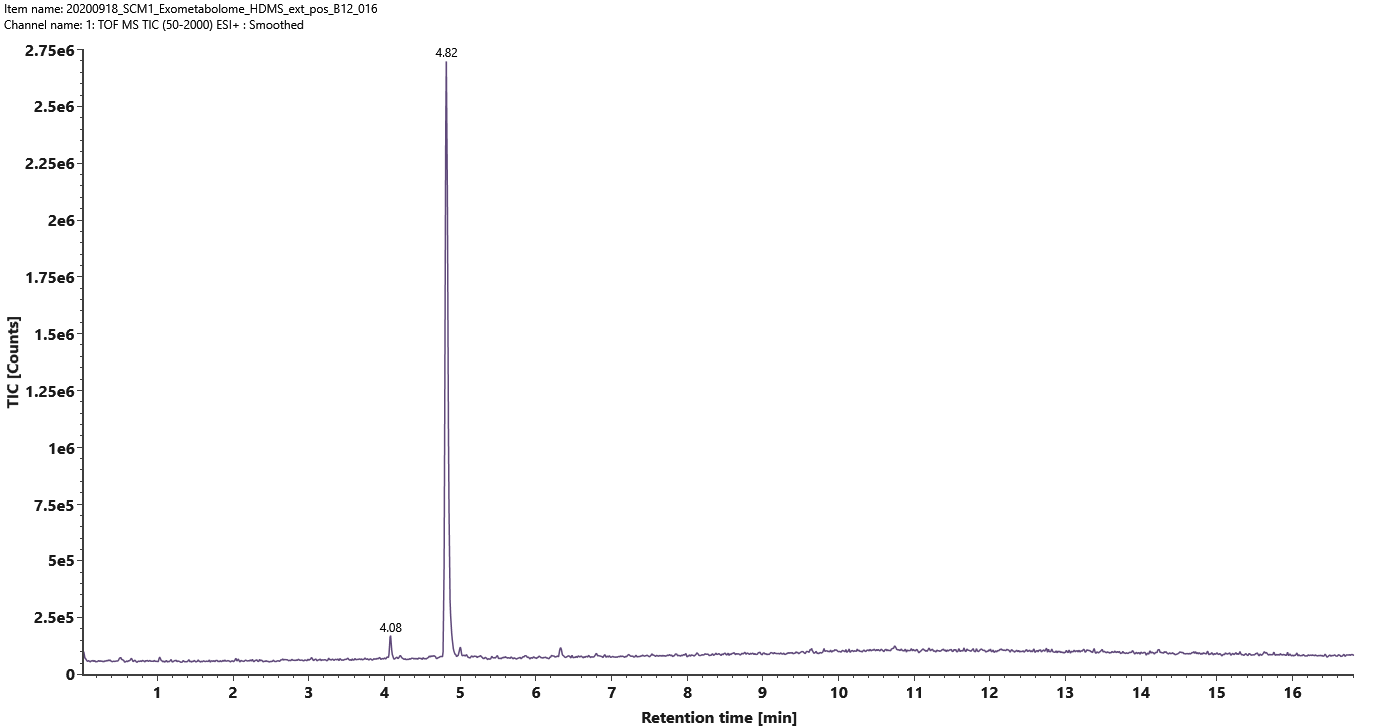


**B**

**A**


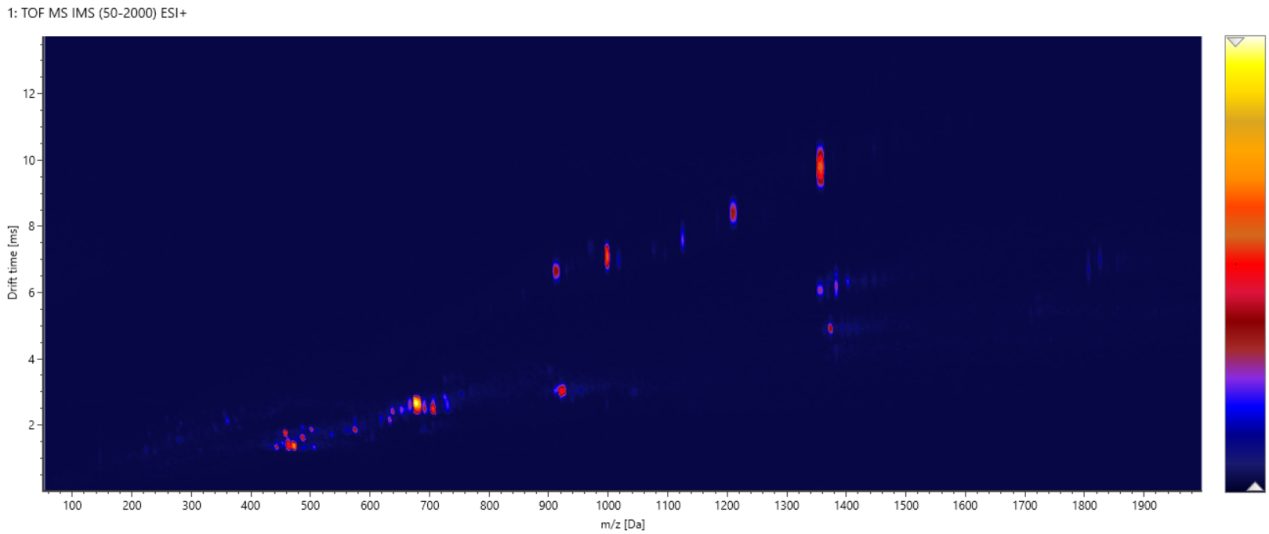


**C**

In-source fragments

In-source fragments

2+

3+

2+

1+

**Supplementary Figure 5.** (A) The ion chromatography, (B) the mass spectrum, and (C) the ion mobility characteristics of Vitamin B_12_ (cobalamin). Data were acquired in the positive ion mode.

**B**

**A**

**Supplementary Figure 6.** CCS-*mz* plots of the spectral features (ions) that are statistically significantly different between the experimental and control mediums. (A) Features or ions that were found higher in the experimental culture mediums (assumed exported extra-cellularly), or (B) higher in the cell-free control mediums (assumed consumed or degraded by extra-cellular enzymes).


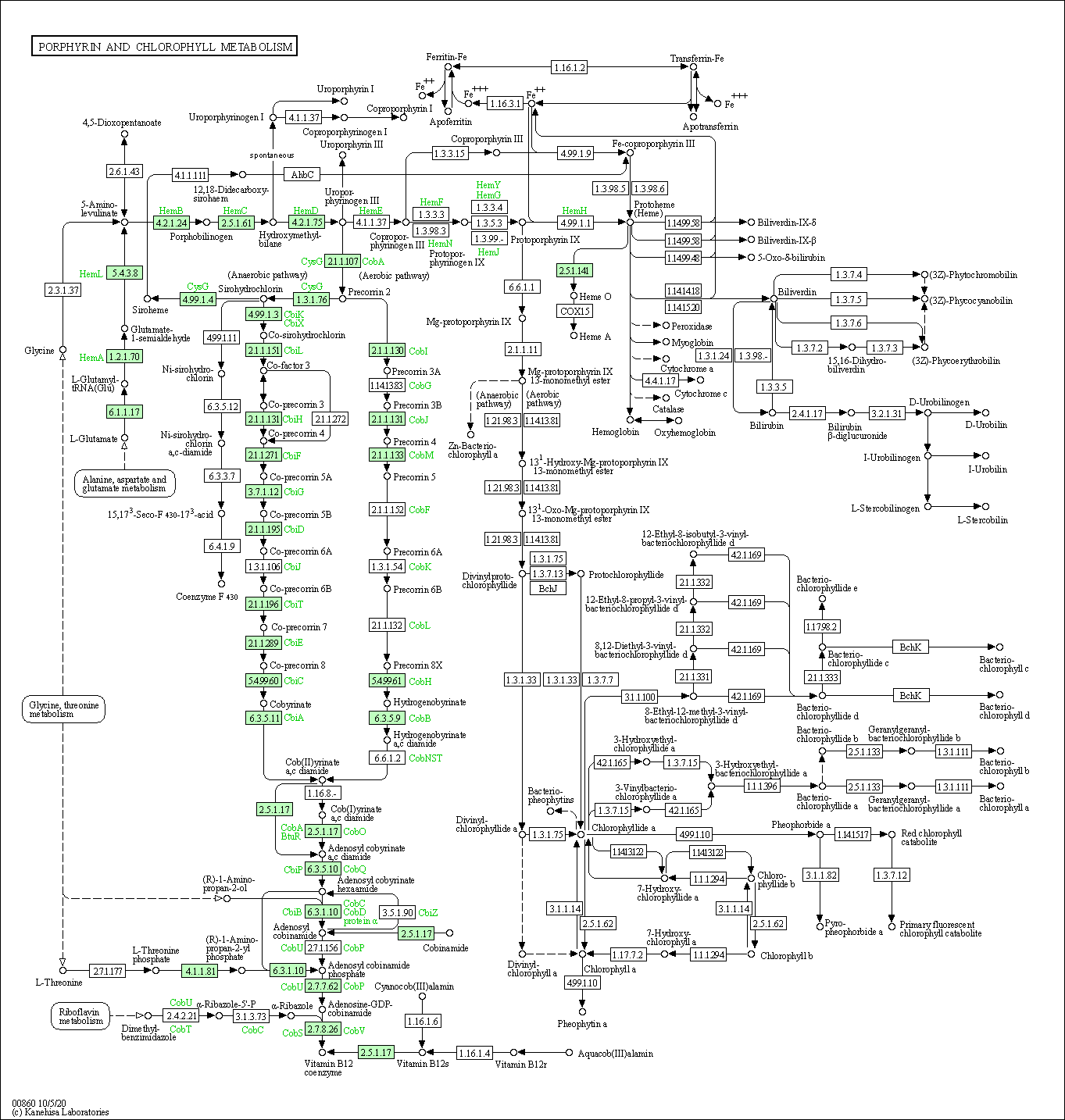


**cobalamin biosynthesis pathway**

**Anaerobic pathway**

**Aerobic pathway**

**Supplementary Figure 7.** Porphyrin and chlorophyll metabolism of *N. maritimus*. Pathway information was obtained from the KEGG database. The filled boxes denote the availability of corresponding genes in the genome of *N. maritimus*. The highlighted sections are the anaerobic cobalamin biosynthesis (highlighted in blue), aerobic cobalamin biosynthesis (highlighted in red), and cobalamin biosynthesis (highlighted in purple) pathways. The final product is Vitamin B_12_ coenzyme (adenosylcobalamin). Because the aerobic pathway is incomplete, it is believed that *N. maritimus* utilizes only the anaerobic pathway for cobalamin biosynthesis.

**A**

Lower axial ligand

(DMB)

α-Ribazole

Cobalamin

Cobinamide

**Cyanocob(III)alamin (Vitamin B_12_)**

**Supplementary Figure 8.** Chemical structure of cyanocob(III)alamin (Vitamin B_12_). Cobalamins consist of a cobalt-containing corrin ring and upper and lower axial ligands. DBM: Dimethylbenzimidazole.


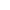

**B**

**A**

**Adenosylcobalamin (Cobamide coenzyme) Methylcobalamin (MeB_12_)**

**C**

**D**

**Hydroxocobalamin cob(I)alamin (Vitamin B_12_s)**

**Supplementary Figure 9.** The upper ligand is exchangeable through both enzymatic and abiotic processes. In biological systems, the upper cyano ligand is replaced by either an adenosyl group, to give adenosylcobalamin, a methyl group, to give methylcobalamin, a water, to give hydroxo or aquacobalamin. or an electron, cob(I)alamin.

**Lower axial ligands**

**Supplementary Figure 10.** Structures of cobamide lower ligands. The lower ligand in contrast is not interchangeable and requires CobT enzymes activation prior to incorporation of the lower ligand into cobamides.
